# SphK1-targeted miR-6784 inhibits functions of skin squamous cell carcinoma cells

**DOI:** 10.18632/aging.202336

**Published:** 2021-01-19

**Authors:** Zhen-Hua Gong, Jiang Ji, Jian Yao, Jian-Feng Ji, Yasu Jiang, Gang Gao, Zhou Feng

**Affiliations:** 1 Department of Plastic and Burn Surgery, The Second Affiliated Hospital of Nantong University, Nantong, China; 2 Department of Dermatology, The Second Affiliated Hospital of Soochow University, Suzhou, China; 3 ENT Department, The Second Affiliated Hospital of Nantong University, Nantong, China; 4 Clinical Laboratory, The Second Affiliated Hospital of Nantong University, Nantong, China

**Keywords:** skin squamous cell carcinoma, SphK1, miR-6784

## Abstract

Sphingosine kinase 1 (SphK1) is overexpressed in skin squamous cell carcinoma (SCC). It has emerged as a novel therapeutic oncotarget. The current study identified a novel SphK1-targeting microRNA, microRNA-6784 (miR-6784). Here, we show that miR-6784 is located at the cytoplasm of A431 skin SCC cells. It directly binds to *SphK1* mRNA. Ectopic overexpression of miR-6784 inhibited *SphK1* 3’-untranslated region (UTR) luciferase activity and downregulated its expression. Moreover, miR-6784 overexpression caused ceramide accumulation in skin SCC cells. Functional studies in established (A431 and SCC9) and primary skin SCC cells revealed that miR-6784 overexpression inhibited cell viability, proliferation, migration, and invasion. It also simultaneously provoked apoptosis activation. Conversely, miR-6784 silencing by antagomiR-6784 induced SphK1 elevation and augmented A431 cell proliferation, migration, and invasion. miR-6784 overexpression-induced anti-A431 cell activity was inhibited by the expression of an UTR-null SphK1 construct. CRISPR/Cas9-induced SphK1 knockout inhibited A431 cell growth. Importantly, miR-6784 was completely ineffective when treating SphK1-knockout A431 cells. Collectively, miR-6784 silences SphK1 and inhibits skin SCC cell progression.

## INTRODUCTION

Skin squamous cell carcinoma (skin SCC) is the second most common skin cancer [1–3], and there is a continuing rise of skin SCC incidence worldwide [1–3]. It is estimated that 180,000 to 400,000 cases of skin SCC would be diagnosed annually in United States [1–4]. Skin SCC usually occurs in skin places exposed to sunlight, *i.e.* head, neck, and ears [1–3]. Sun exposure and immunosuppression are two primary risk factors for skin SCC [1–3], as long-term exposure to the sun is the most significant environmental risk factor [1–3], while skin SCCs at lips and ears have a high rate of local recurrence and distant metastasis [1–3]. Exploring novel molecularly-targeted therapies is vital for more efficient anti-skin SCC treatments [1–3].

Sphingosine kinase 1 (SphK1) is a primary member of SphK family proteins (the other one is SphK2) [5]. It phosphorylates sphingosine to sphingosine-1-phosphate (S1P) [6–9], an important signaling lipid messenger with both intracellular and extracellular functions [6–9]. Intracellular S1P accumulation promotes cell proliferation and survival [10, 11]. S1P is also a ligand for endothelial differentiation gene 1 (EDG1) [6–9]. Various stimuli would increase cellular S1P contents by activating SphK1 [6–9]. Conversely, SphK1 inhibition or silencing should block S1P formation, and cause the accumulation of pro-apoptotic ceramide and sphingosine [6–9]. The change of these lipid messengers would eventually cause proliferation arrest and cell apoptosis [6–9].

Studies have shown that SphK1 expression is elevated in skin SCC tissues and cells [12, 13]. It has emerged as an important prognostic marker and valuable therapeutic target for skin SCC [12, 13]. SphK1 overexpression is vital for skin SCC cell proliferation, migration, and metastasis [12, 13]. SphK1 inhibition, however, would lead to ceramide production that promotes cancer cell apoptosis [12, 13]. The anti-skin SCC activity by I-BET726, a novel bromodomain-containing protein 4 (BRD4) inhibitor, has been associated with SphK1 inhibition and ceramide accumulation [14]. These results indicated that SphK1 inhibition or silencing should produce significant anti-skin SCC activity.

MicroRNAs (miRNAs) are small non-coding RNAs (ncRNAs) that change gene expression at both translational and post-transcriptional levels [15–18]. The 21-25 nucleotide miRNAs bind to the 3’ untranslated region (3’-UTR) of the complementary mRNAs, and leads to translation inhibition and/or degradation of targeted mRNAs [15–18]. Dysregulation of miRNA, which is associated with tumorigenesis and cancer progression, has become a characteristic marker of skin SCC [19–21]. Silencing SphK1 expression with specific miRNAs has proven to be an appropriate strategy to produce significant anti-cancer cell activity [22–25]. Here, we discovered microRNA-6784 (miR-6784) as a novel SphK-targeting miRNA. Furthermore, miR-6784 was able to silence SphK1 and inhibit skin SCC cell progression.

## RESULTS

### miR-6784 binds to and silences SphK1 in skin SCC cells

**Figure 1:**
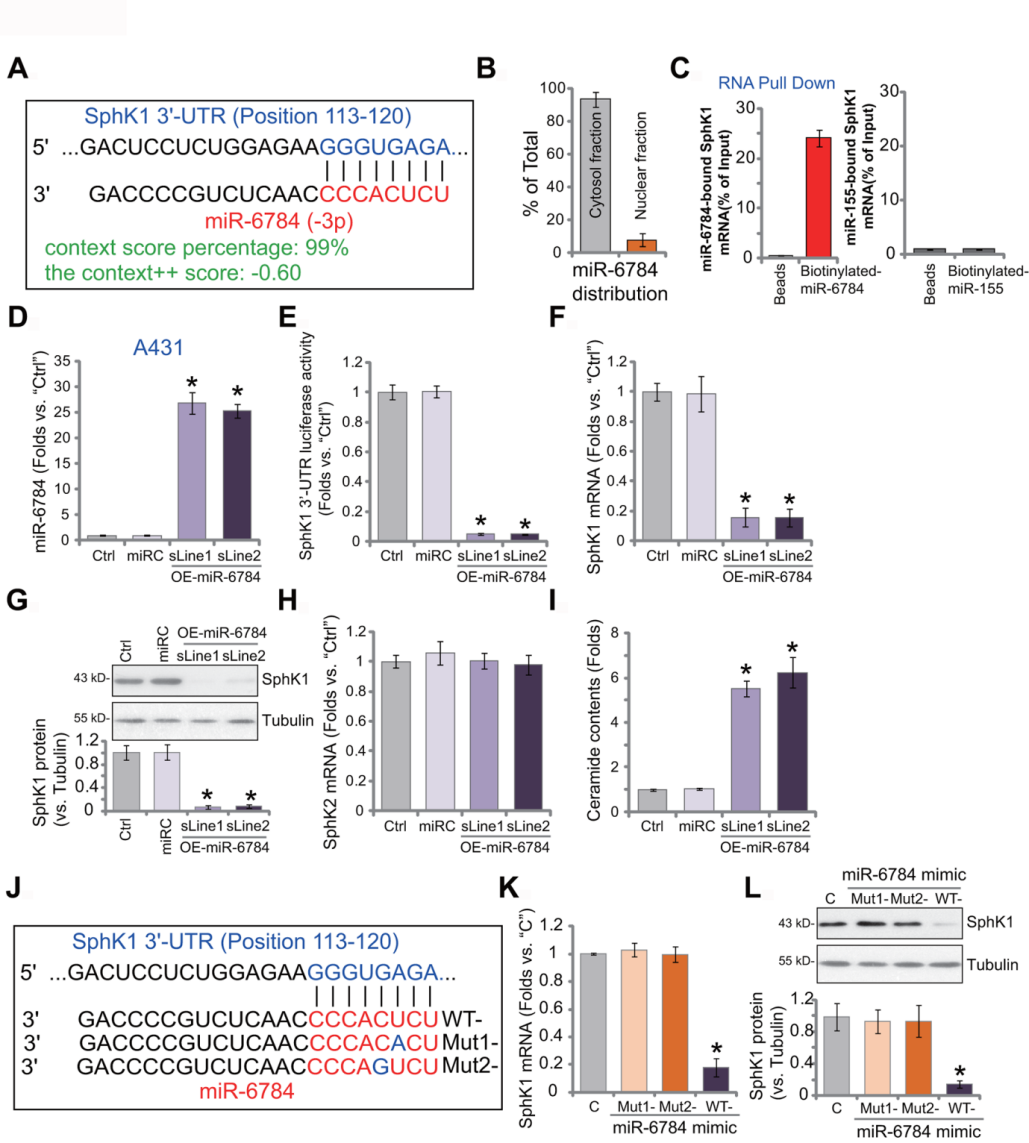
**miR-6784 binds to and silences SphK1 in skin SCC cells.** microRNA-6784 (miR-6784) putatively targets *SphK1* 3’-UTR at position 113-120 (**A**). The subcellular localization of miR-6784 was tested by qPCR, with results normalized to total miR-6784 level (**B**). Association between the biotinylated-miR-6784 or the biotinylated-miR-155 with *SphK1* mRNA was tested by RNA-Pull Down assays in A431 cells (**C**). Stable A431 cells expressing the lentiviral construct encoding pre-miR-6784 (“OE-miR-6784-sLine1/2”, two lines) or the nonsense control microRNA sequence (“miRC”) were established. Expression of miR-6784, SphK1 and SphK2 was examined (**D**, **F**, **G**, **H**), with the relative *SphK1* 3’-UTR luciferase activity tested as well (**E**). Cellular ceramide contents were shown (**I**). A431 cells were transfected with 500 nM of wild-type (WT-) or mutant miR-6784 mimics (“Mut1-/-2”, sequences listed in **J**), and the control cells were transfected with nonsense control miRNA mimic (“C”); After 48h, *SphK1* mRNA (**K**) and protein (**L**) expression was tested. Data were presented as mean ± standard deviation (SD, n=5). Experiments in this study were repeated three times with similar results obtained. *p< 0.05 *vs.* “miRC”/“C” cells.

First, the miRNA database TargetScan (V7.2) [26] was utilized to search possible miRNAs that can target the *3’-UTR* of SphK1. The candidate miRNAs with high binding scores to *SphK1* mRNA were further verified in other miRNA databases. The bioinformatic studies discovered one particular miRNA, miR-6784, which putatively binds to *SphK1*3’-UTR (at position 113-120) (Figure 1A). The binding context score percentage is 99%, and the context^++^ score is -0.60 (Figure 1A). These parameters indicated a high percentage of possible direct binding between miR-6784 and *SphK1* 3’-UTR [26]. When analyzing subcellular localization of miR-6784, we found that over 92% of endogenous miR-6784 was located at the cytosol fraction of A431 cells (Figure 1B). Only less than 8% was located at the nuclear fraction (Figure 1B). By applying a RNA Pull Down assay, we found that the biotinylated-miR-6784 can directly associate with *SphK1* mRNA in A431 cells (Figure 1C). As a negative control, biotinylated-miR-155 failed to bind to *SphK1* mRNA in A431 cells (Figure 1C).

To test whether miR-6784 could change the expression of SphK1, A431 cells were transfected with lentivirus encoding pre-miR-6784 (lv-miR-6784). Following selection by puromycin-containing medium, two stable A431 cell lines were established: OE-miR-6784-sLine1 and OE-miR-6784-sLine2. As shown, in OE-miR-6784 A431cells, mature miR-6784 (-3p) level increased over 25 folds (*vs.* control cells) (Figure 1D). In contrast, *SphK1* 3-UTR luciferase reporter activity robustly decreased (Figure 1E). In the OE-miR-6784 A431 cells, *SphK1* mRNA expression was downregulated (Figure 1F). Furthermore, SphK1 protein silencing was detected in miR-6784-overexpresssed A431 cells (Figure 1G). miR-6784 overexpression did not alter *SphK2* mRNA expression in A431 cells (Figure 1H). It, however, induced ceramide accumulation (Figure 1I). The nonsense control miRNA, miRC, did not alter miR-6784-SphK1/2 expression nor the ceramide contents (Figure 1D–1I).

Next, we created two mutant miR-6784 mimics with mutations at the binding sites of *SphK1* 3’-UTR (sequences listed in Figure 1J). These mutant miR-6784 mimics or the wild-type (WT-) miR-6784 mimic were separately transfected into A431 cells. As shown, only WT-miR-6784 mimic downregulated *SphK1* mRNA (Figure 1K) and protein (Figure 1L). The two mutants were ineffective (Figure 1K, 1L). Therefore, miR-6784 targets and silences *SphK1* in A431 cells.

### miR-6784 overexpression exerts significant anti-skin SCC cell activity

**Figure 2:**
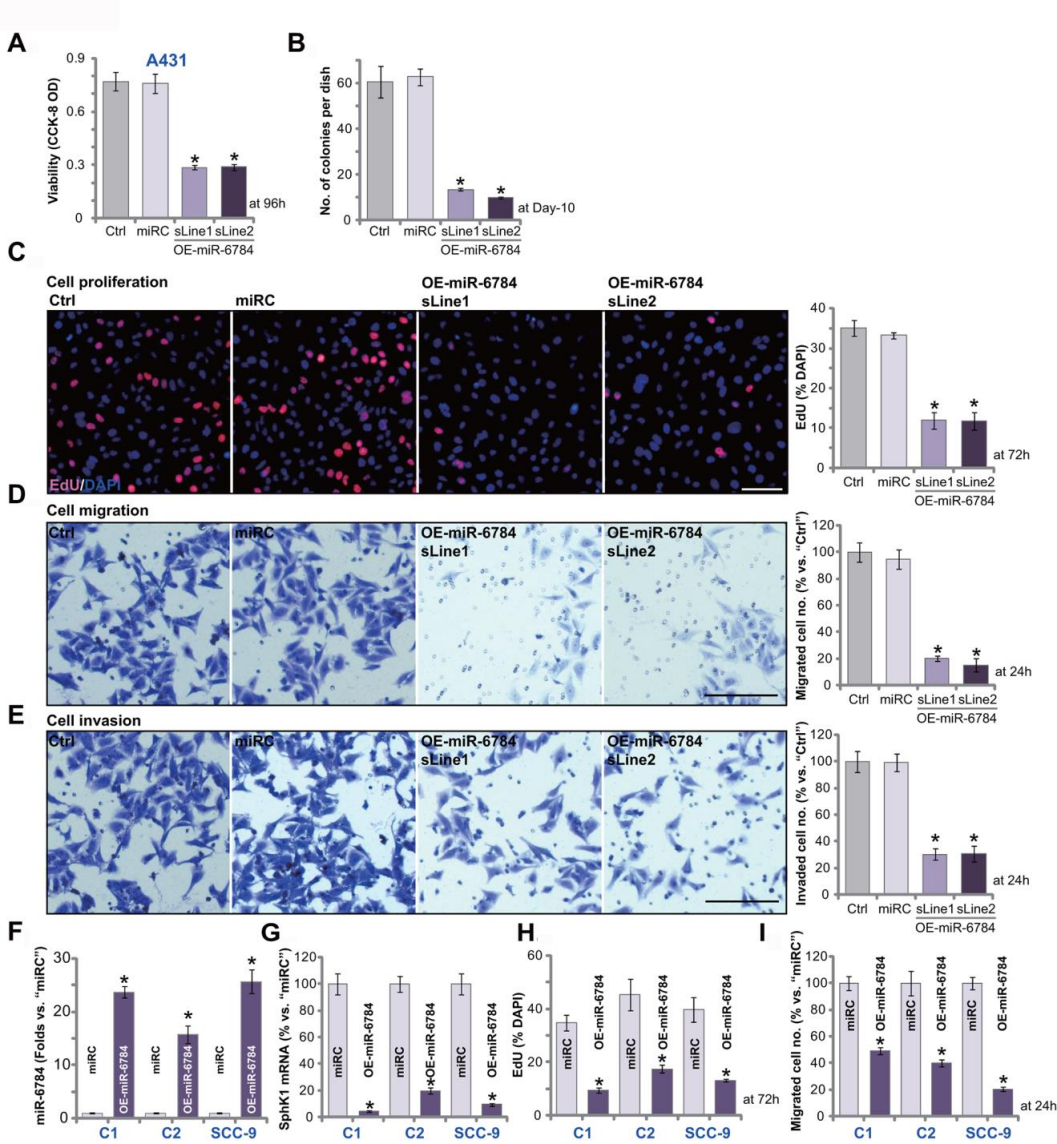
**miR-6784 overexpression exerts significant anti-skin SCC cell activity.** A431 cells (**A**–**E**), primary skin SCC cells (“C1”/“C2”, **F**–**I**), SCC-9 cells (**F**–**I**) expressing the lentiviral construct encoding pre-miR-6784 (“OE-miR-6784”) or the nonsense control microRNA sequence (“miRC”), were established. Cells were cultured for applied time periods, cellular functions, including cell viability (**A**), colony formation (**B**), cell proliferation (**C**, **H**), migration (**D**, **I**), and invasion (**E**) were tested by the appropriate assays. Expression of miR-6874 (**F**), *SphK1* mRNA (**G**) in “C1”/“C2” cells, and SCC-9 cells was examined as well. Data were presented as mean ± standard deviation (SD, n=5). Experiments in this study were repeated three times with similar results obtained. *p< 0.05 *vs.* “miRC” cells. Scale bar=100 μm (**C**–**E**).

Next, we studied the potential function of miR-6784 in skin SCC cells. CCK-8 is a well-established assay to study cell viability. As shown, in miR-6784-overexpressed A431 cells OE-miR-6784-sLine1 and OE-miR-6784-sLine2 (see Figure 1), CCK-8 viability OD was significantly reduced (*vs.* control cells, Figure 2A). The number of viable cell colonies was decreased as well in OE-miR-6784 A431 cells (Figure 2B). Furthermore, miR-6784 overexpression inhibited EdU incorporation in A431 cells (Figure 2C), indicating proliferation inhibition. Cell migration and invasion were tested by “Transwell” and “Matrigel Transwell” assays, respectively. Results demonstrated that ectopic miR-6784 overexpression largely suppressed A431 cell migration (Figure 2D) and invasion (Figure 2E). As expected, the nonsense control microRNA sequence, or miRC, did not alter A431 cell functions (Figure 2A–2E).

Whether miR-6784 exerted similar effects in other skin SCC cells was tested. The primary human skin SCC cells derived from two skin SCC patients (namely “C1/C2”, from Dr. Liu [14, 27]), as well as SCC-9 established cells, were utilized. These cells were transfected with the lentivirus encoding pre-miR-6784 (lv-miR-6784). Stable OE-miR-6784 cells were established with puromycin selection. When compared to the control cells (with miRC), levels of mature miR-6784 were upregulated in OE-miR-6784 cells (Figure 2F), where *SphK1* mRNA expression was silenced (Figure 2G). In “C1” and “C2” primary cells and SCC-9 cells, miR-6784 overexpression inhibited cell proliferation (decreased EdU-positive nuclei ratio, Figure 2H) and migration (“Transwell” assays, Figure 2I). Therefore, in skin SCC cells, miR-6784 overexpression inhibited cell survival, proliferation, migration, and invasion.

### miR-6784 overexpression induces apoptosis activation in skin SCC cells

**Figure 3:**
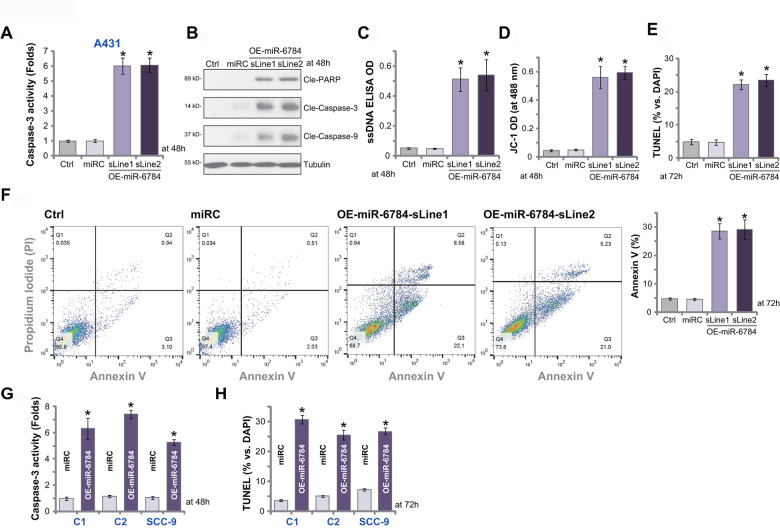
**miR-6784 overexpression induces apoptosis activation in skin SCC cells.** A431 cells (**A**–**F**), primary skin SCC cells (“C1”/“C2”, **G**, **H**), and SCC-9 cells (**G**, **H**) expressing the lentiviral construct encoding pre-miR-6784 (“OE-miR-6784”) or the nonsense control microRNA sequence (“miRC”) were established. Cells were cultured for applied time periods, caspase and apoptosis activation were tested by mentioned appropriate assays. Data were presented as mean ± standard deviation (SD, n=5). Experiments in this study were repeated three times with similar results obtained. *p< 0.05 *vs.* “miRC” cells.

SphK1 silencing or inhibition would lead to ceramide accumulation and cell apoptosis [5]. Since miR-6784 overexpression silenced SphK1 and induced ceramide accumulation (see Figure 1), we tested its effect on cell apoptosis. The relative caspase-3 activity was significantly increased in miR-6784-overexpressed A431 cells, OE-miR-6784-sLine1, and OE-miR-6784-sLine2 (Figure 3A). Furthermore, in A431 cells miR-6784 overexpression induced cleavages of caspase-3, caspase-9, and poly (ADP -ribose) polymerase (PARP) (Figure 3B). In addition, it increased the accumulation of single strand DNA (ssDNA) (indicating DNA breaks, Figure 3C). With the evidence that JC-1 green monomers accumulated (increased intensity) in the mitochondria, we confirmed mitochondrial depolarization in OE-miR-6784 A431 cells (Figure 3D). These results indicated the activation of caspase-dependent mitochondrial apoptosis pathway in miR-6784-overexpressed A431 cells [28, 29].

In OE-miR-6784 A431 cells, the TUNEL-positive nuclei ratio was significantly increased (Figure 3E) indicating cell apoptosis induction. It was further confirmed by FACS assays, which showed the increased number of Annexin V-gated A431 cells (Figure 3F). The nonsense control microRNA, miRC, failed to induce significant caspase and apoptosis activation in A431 cells (Figure 3A–3F). In “C1” and “C2” primary cells and SCC-9 cells, ectopic miR-6784 overexpression (“OE-miR-6784”, see Figure 2) significantly increased caspase-3 activity (Figure 3G) and TUNEL-positive nuclei ratio (Figure 3H), suggesting apoptosis activation. These results suggested that miR-6784 overexpression provoked apoptosis activation in skin SCC cells.

### miR-6784 inhibition induces SphK1 elevation and promotes A431 cell progression

**Figure 4:**
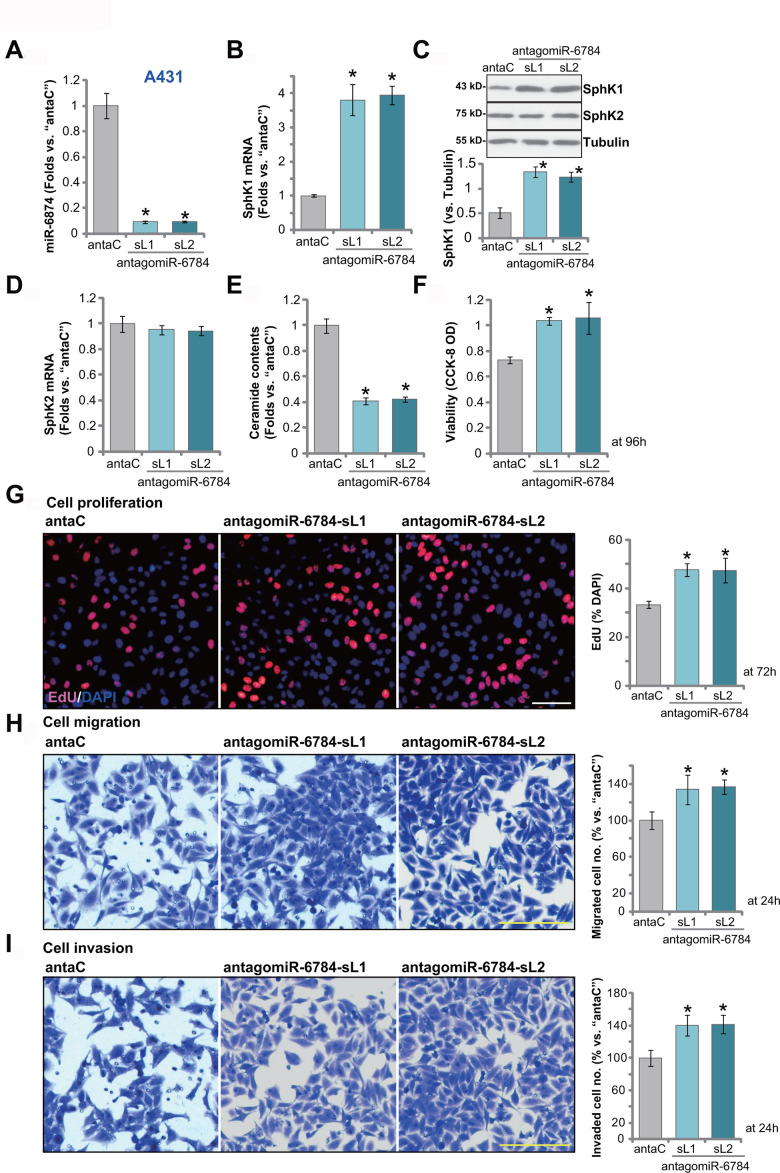
**miR-6784 inhibition induces SphK1 elevation and promotes A431 cell progression.** A431 cells, expressing the lentiviral construct encoding the pre-miR-6784 anti-sense (“antagomiR-6784-sL1/sL2”, two stable cell lines) or the control nonsense microRNA anti-sense (“antaC”), were established. Cells were cultured for applied time periods. Expression miR-6784, SphK1 and SphK2 (**A**–**D**) was tested; Cellular ceramide contents were shown (**E**); Cell viability (CCK-8 OD, **F**), proliferation (by recording EdU-positive nuclei ratio, **G**), migration (“Transwell” assay, **H**), and invasion (“Matrigel Transwell” assay, **I**) were tested. Data were presented as mean ± standard deviation (SD, n=5). Experiments in this study were repeated three times with similar results obtained. *p< 0.05 *vs.* “antaC” cells. Scale bar=100 μm (**G**–**I**).

Results have demonstrated that miR-6784 overexpression silenced SphK1 and exerted anti-skin SCC cell activity. We therefore hypothesized that miR-6784 inhibition would exert opposite functions. To test this hypothesis, A431 cells were transduced with a lentiviral construct encoding the antisense of pre-miR-6784 (antagomiR-6784). Two stable cell lines were established following puromycin selection: antagomiR-6784-sL1 and antagomiR-6784-sL2. When compared to the control A431 cells with control miRNA anti-sense (“antaC”), the mature miR-6784 expression downregulated over 90% in antagomiR-6784-expressing A431 cells (Figure 4A). Consequently, *SphK1* mRNA expression was upregulated (Figure 4B). SphK1 protein expression was elevated as well (Figure 4C). Expressions of *SphK2* mRNA (Figure 4D) and protein (Figure 4C) were however unchanged. Cellular ceramide contents were decreased in antagomiR-6784-expressing A431 cells (Figure 4E).

CCK-8 assay results demonstrated that miR-6784 inhibition by antagomiR-6784 increased the viability of A431 cells (Figure 4F). EdU-positive nuclei ratio was increased as well in the antagomiR-6784-expressing cells (*vs.* antaC control cells, Figure 4G). Furthermore, tested by “Transwell” and “Matrigel Transwell” assays, antagomiR-6784 augmented A431 cell migration (Figure 4H) and invasion (Figure 4I). These results demonstrated that miR-6784 inhibition induced SphK1 elevation and promoted skin SCC cell progression.

### miR-6784-induced anti-A431 cell activity is due to SphK1 silencing

**Figure 5:**
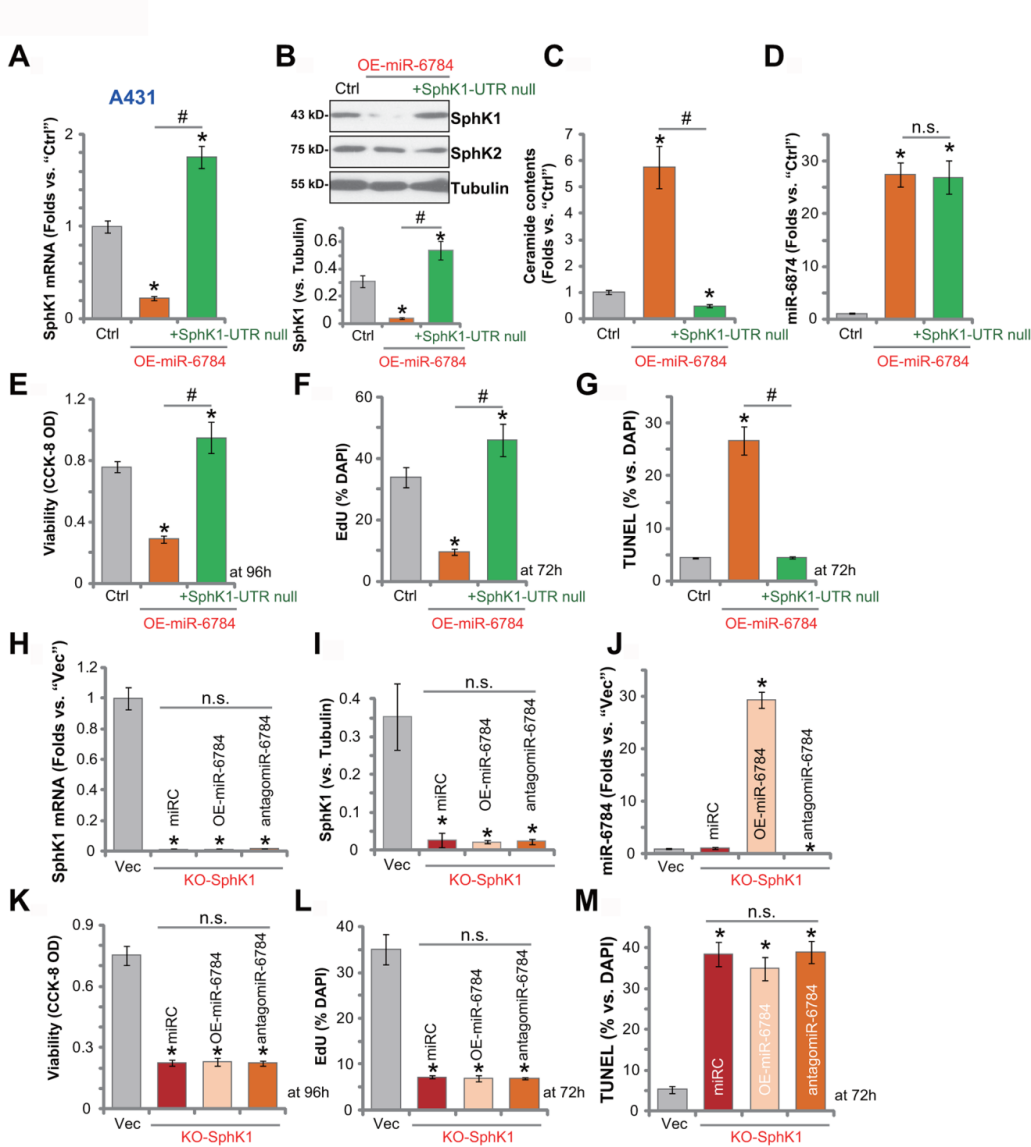
**miR-6784-induced anti-A431 cell activity is due to SphK1 silencing.** The miR-6784-overexpressed A431 cells (OE-miR-6784-sLine1) were further transduced with or without an UTR-null SphK1 construct (“+SphK1-UTR null”), and stable cells were established with puromycin selection; Control cells were the parental control cells (“Ctrl”); Expression SphK1/2 and miR-6784 was shown (**A**, **B**, **D**); Cellular ceramide contents were tested (**C**); Cells were further cultured for applied time periods; Cell viability, proliferation, and apoptosis were tested by CCK-8 (**E**), EdU staining (**F**), and TUNEL staining (**G**) assays, respectively. Stable A431 cells with the CRISPR-Cas9-SphK1-KO-GFP construct (“KO-SphK1”) were further infected with lv-antagomiR-6784 (“antagomiR-6784”), lv-pre-miR-6784 (“OE-miR-6784”), or nonsense control miRNA sequence (“miRC”) for 48h, and puromycin was added to select stable cells. Control cells were transduced with empty vector (“Vec”); Expressions of SphK1 and miR-6784 were shown and results were quantified (**H**–**J**); Cells were further cultured for applied time periods, cell viability (**K**), proliferation (**L**), and apoptosis (**M**) were tested similarly. Data were presented as mean ± standard deviation (SD, n=5). Experiments in this study were repeated three times with similar results obtained. *p< 0.05 *vs.*“Ctrl”/“Vec” cells. ^#^*p*< 0.05. “n.s.” stands for non-statistical difference.

To test whether miR-6784-induced anti-skin SCC cell activity was due to SphK1 silencing, an UTR-null SphK1 construct (“SphK1-UTR null”, from Dr. Yao [22]) was transduced to miR-6784-overexpressed A431 cells (OE-miR-6784-sLine1, see Figure 1–3). Here, the SphK1-UTR null construct restored *SphK1* mRNA (Figure 5A) and protein (Figure 5B) expression in OE-miR-6784 cells. Its expression level was even higher than that in the control cells (Figure 5A, 5B). Though, the protein expression of SphK2 was unchanged (Figure 5B). The SphK1-UTR null construct inhibited ceramide accumulation in OE-miR-6784 cells (Figure 5C), however it failed to alter the miR-6784 expression (Figure 5D).

Functional studies demonstrated that miR-6784 overexpression induced viability (CCK-8 OD) reduction (Figure 5E), proliferation inhibition (by recording EdU-positive nuclei ratio, Figure 5F), and cell apoptosis (by recording TUNEL-positive nuclei ratio, Figure 5G), which were all largely inhibited by the SphK1-UTR null construct. Thus, restoring SphK1 expression inhibited miR-6784 overexpression-induced anti-A431 cell activity. These results suggested that SphK1 silencing might be the primary mechanism of miR-6784-induced actions in skin SCC cells.

In A431 cells, transfection of UTR-null SphK1 construct (“SphK1-UTR null”) alone resulted in significantly elevated expression of *SphK1* mRNA and protein (Supplementary Figure 1A). As a result, ceramide contents were decreased (Supplementary Figure 1B) with unchanged miR-6784 expression (Supplementary Figure 1C). Mimicking antagomiR-6784-induce actions by adding SphK1-UTR null construct increased A431 cell viability (Supplementary Figure 1D) and promoted cell proliferation (Supplementary Figure 1E). Cell apoptosis, as expected, was not induced by this construct (Supplementary Figure 1F).

We further hypothesized that SphK1 depletion should mimic the activity induced by miR-6784 overexpression in skin SCC cells. Therefore, the CRISPR/Cas9 strategy was utilized to knockout SphK1 in A431 cells using a lentiCRISPR-SphK1-KO plasmid (from Dr. Yao [22]). Then KO-SphK1 stable cells with depleted *SphK1* mRNA (Figure 5H) and protein (see quantified results in Figure 5I) were established. SphK1 KO in A431 cells did not change miR-6784 expression (Figure 5J). However, it induced significant viability reduction (Figure 5K), proliferation inhibition (EdU assay, Figure 5L), and cell apoptosis (TUNEL assay, Figure 5M). Importantly, in KO-SphK1 A431 cells, exogenously altering miR-6784 expression by lv-miR-6784 (OE-miR-6784) or antagomiR-6784 (Figure 5J) did not alter cell viability (Figure 5K), proliferation (Figure 5L), or apoptosis (Figure 5M). These results indicated that miR-6784 did not change the activities of SphK1 KO A431 cells, further supporting that SphK1 is the primary target of miR-6784 in skin SCC cells.

## DISCUSSION

Sphingolipids, including ceramide, S1P, and sphingosine, are key signaling lipids that are important for cancer pathogenesis and progression [30–33]. These sphingolipids can therefore influence cancer cell biological outcomes including cell survival, differentiation, apoptosis, migration, and proliferation [30–33]. Activated SphK1 catalyzes the conversion of sphingosine and ceramide to S1P, thus help achieving the cancer-promoting outcomes [30–33]. Contrarily, SphK1 silencing or inhibition would lead to ceramide accumulation, proliferation arrest, and cell apoptosis [30–33].

Studies have shown that skin SCC and various other types of human cancers acquire survival advantage, hyper-proliferative properties, aggressiveness, and chemotherapy resistance via non-oncogenic addiction to S1P signaling [30–33]. In oral SCC cells, downregulation of leucine-rich repeats and immunoglobulin-like domains 1 (LRIG1) induced the activation of EGFR-mediated SphK1 signaling to promote extracellular matrix (ECM) remodeling and cancer cell progression [34]. *Tamashiro* et al., showed that SphK1 was required for the invasion of head and neck SCC (HNSCC) cells [35]. Furthermore, SphK1 inhibition sensitized radiation-induced anti-HSNCC cell activity [36]. These results clearly indicated that SphK1 is a promising therapeutic target for skin SCC.

SphK1 can be targeted and silenced by miRNAs in various cancer cells to produce significant anti-cancer cell activity [22–25]. In gastric cancer cells, miR-124 targeted and silenced SphK1 to inhibit proliferation [25]. Ovarian cancer cell migration was inhibited by the SphK1-targeting miR-124 [24]. By silencing SphK1, miR-124 inhibited proliferation and invasion of osteosarcoma (OS) cells [23]. Yao et al., discovered miR-6784 as a novel SphK1-targeting miRNA, which silenced SphK1 to induce OS cell apoptosis [22]. In human bladder cancer cells, miR-125b silenced SphK1 and inhibited cell proliferation and migration [37]. Similarly, miR-506 downregulation increased SphK1 expression and promoted pancreatic cancer progression [38]. Therefore, miR-mediated silencing of SphK1 could efficiently inhibit progression of various types of cancer cells.

To our knowledge miR-6784 is a relatively novel miRNA with its functions largely unknown. Here, we found that miR-6784 is a novel SphK-targeting miRNA in skin SCC cells. miR-6784 is located at the cytoplasm of skin SCC cells and it binds directly to *SphK1* mRNA. In established and primary skin SCC cells, SphK1’s 3-UTR activity, as well as its mRNA and protein expression, decreased following the overexpression of miR-6784. Conversely, antagomiR-6784-mediated miR-6784 silencing elevated *SphK1* mRNA and protein. Importantly, the mutant miR-6784 mimics, with mutations at the SphK1’s 3-UTR-binding sites, failed to inhibit SphK1 expression. Therefore, miR-6784 directly binds to and silences SphK1 in skin SCC cells.

In established (A431 and SCC9) and primary skin SCC cells, ectopic miR-6784 overexpression inhibited cell viability, proliferation, migration, and invasion. Moreover, it provoked significant apoptosis activation. As miR-6784 silenced by antagomiR-684, SphK1 elevation would be induced to promote A431 cell proliferation, migration, and invasion. Therefore, miR-684-induced SphK1 silencing provoked robust anti-skin SCC cell activity.

The results of this study indicated that SphK1 silencing should be the primary mechanism of miR-6784 in skin SCC cells. To restore SphK1 expression by an UTR-null SphK1 construct, completely reversed miR-6784-induced actions. CRISPR/Cas9-induced SphK1 KO also resulted in proliferation inhibition and apoptosis in A431 cells. Importantly, miR-6784 overexpression or silencing was ineffective in SphK1-KO A431 cells.

## CONCLUSIONS

Nowadays, over one million cases of skin SCC are diagnosed each year as SCC incidence has increased up to two folds in the past three decades [1–3]. The advanced, recurrent, and metastatic skin SCC are still fatal to many affected patients [1–3]. Targeted molecular therapies are vital for better skin SCC treatments [1–3]. The results of this study showed that miR-6784 silenced SphK1 and inhibited skin SCC cell progression. Therefore, miR-6784 could be a novel therapeutic candidate for skin SCC.

## MATERIALS AND METHODS

### Chemicals, reagents and antibodies

Puromycin, polybrene, CCK-8 viability kit, and cell culture reagents were purchased from Sigma-Aldrich (St. Louis, MO, USA). Antibodies were purchased from Cell Signaling Tech (Beverly, MA, USA). Lipofectamine 3000, TUNEL (terminal deoxynucleotidyl transferase dUTP nick end labeling) apoptosis assay kit, Annexin V, and propidium iodide (PI) assay kit were provided by Thermo-Fisher Invitrogen (Carlsbad, CA, USA). Primers, viral constructs, and sequences were provided by Genechem (Shanghai, China) unless otherwise mentioned.

### Cell culture

Established skin SCC cell lines, A431 and SCC9, were provided by Dr. Liu at Wenzhou Medical University [14, 27]. The primary skin SCC cells derived from two written-informed consent SCC patient (“C1/C2”, with PTEN depletion and p53-null) were provided by Dr. Liu [14] as well. Cells were cultured as reported before [39]. Protocols were approved by the Ethics Committee of Nantong University, in accordance with Declaration of Helsinki.

### Quantitative real time-PCR (qPCR)

As reported [22, 40], TRIzol reagents (Thermo Fisher Scientific, Shanghai, China) were utilized to extract total cellular RNA that was reversely transcripted [41]. An ABI Prism 7500 system was applied to perform qPCR using a SYBR GREEN PCR Master Mix (Thermo Fisher Scientific). The product melting temperature was always calculated. *Glyceraldehyde-3-phosphatedehydrogenase*(*GAPDH)* was tested as the internal control and reference gene for data quantification using an established 2^−ΔΔ*C*t^ method. Expression of miR-6784 was normalized to U6. The primers of this study were listed in Table 1.

**Table 1:** Sequences in this study.

**Genes**	**Forward Sequence (5’-3’)**	**Reverse Sequence (5’-3’)**
*miR-6784*	GCCGGGGCTTTGGGTGAG	GAACATGTCTGCGTATCTC
*U6 RNA*	CTCGCTTCGGCAGCACAT	TTTGCGTGTCATCCTTGCG
*SphK1*	GCTGGCAGCTTCCTTGAACCAT	GTGTGCAGAGACAGCAGGTTCA
*SphK2*	GAGGAAGCTGTGAAGATGCCTG	GAGCAGTTGAGCAACAGGTCGA
*GAPDH*	GTCTCCTCTGACTTCAACAGCG	ACCACCCTGTTGCTGTAGCCAA

**qPCR primers miR-6784** Precursor:

UACAGGCCGGGGCUUUGGGUGAGGGACCCCCGGAGUCUGUCACGGUCUCACCCCAACUCUGCCCCAG

Mature miR-6784 (-3p) sequence: GCCGGGGCUUUGGGUGAGGG

### Western blotting

As reported before [42, 43], total cellular protein lysates (40 μg proteins per treatment into each lane) were separated by 10-12% SDS-PAGE gels and then transferred to polyvinylidene fluoride (PVDF) membranes (Sigma-Aldrich, St. Louis, MO, USA). The membranes were blocked and immuno-blotted with indicated primary and secondary antibodies. An enhanced chemiluminescence (ECL) reagent kit (Bio-Rad, Shanghai, China) was utilized to visualize interested protein bands. The ImageJ software (NIH) was utilized for data quantification.

### Forced miR-6784 overexpression or inhibition

The protocol was based on a previous study [22]. A lentiviralGV369 construct encoding the miR-6784 precursor (pre-miR-6784) (sequence listed in Table 1) and a GV369 construct encoding the pre-miR-6784 anti-sense sequence (antagomiR-6784) were synthesized by Genechem Co. (Shanghai, China). Each construct was transfected to HEK-293 cells along with lentivirus package constructs (psPAX2 and pMD2.G [44], Genechem). Thereafter, the pre-miR-6784-expressing lentivirus (“lv-pre-miR-6784”) or the anti-sense lentivirus (“lv-antagomiR-6784”) were established. After enrichment, lentivirus was added to skin SCC cells (cultured in polybrene-containing complete medium, at 50-60% confluence). To select stable cells, puromycin (3.0 μg/mL) was added in the complete medium for 5-6 passages. In stable cells, mature miR-6784 (“-3p”, sequence listed in Table 1) expression was always examined by qPCR assays.

### Transfection of miR mimic

A431 cells were initially seeded into six-well plates (1×10^5^ cells per dish) and transfected with the applied miR mimic (500 nM × two rounds, each for 24h) using Lipofectamine 3000 [45].

### SphK1 3'-UTR activity assay

As reported before [22], the firefly-luciferase reporter vector pGL4.13 (luc2/SV40) with the *SphK1* 3’-UTR containing miR-6784’s putative binding sites (at position 113-120) was provided by Dr. Yao at Nanjing Medical University [22]. A431 cells were initially seeded into six-well plates (1×10^5^ cells per dish) and transfected with the plasmid, Renillaluciferase reporter vector, and pRL-SV40 (Promega, Madison, WI, USA ) [22] with Lipofectamine 3000. Cells were then infected with the applied miR-6784 lentivirus with SphK13'-UTR luciferase, and the activity was examined through a Promega kit [45].

### RNA-Pull down assay

The detailed protocol for RNA-Pull Down assay via a Pierce Magnetic RNA Pull-Down Kit has been described previously [46, 47]. Briefly, A431 cells were transfected with biotinylated miR-6784 mimic or control mimic (Genechem, 200 nmol/L) for 24h, and the cells were harvested [47]. The quantified total cellular lysates were incubated with streptavidin-coated magnetic beads to pull-down biotin-captured RNA complex that was purified [46]. *SphK1 mRNA* was examined through qPCR and normalized to the input controls.

### CCK-8 viability assay

Cells with applied genetic modifications were seeded into 96-well plates (exact 4, 000 cells per well) and cultured for 96h. Cell viability was examined by the CCK-8 assay kit, and in each well CCK-8’s, optical density (OD) was tested at 450 nm.

### Colony formation

A431 cells with applied genetic modifications were initially seeded into 10-cm dishes (exact 2×10^5^ cells per dish). Complete medium was renewed every two days for a total of 10 days. Afterwards, the number of viable A431 colonies was manually counted.

### EdU (5-ethynyl-20-deoxyuridine) assay

As reported [14], cells with applied genetic modifications were initially seeded into six-well plates (1×10^5^ cells per well) and cultured for 72h. Afterwards, an EdU Apollo-567 assay kit (RiboBio, Guangzhou, China) was utilized to stain proliferative nuclei (EdU-positive). Five random views under a fluorescent microscope (1000 nuclei per treatment) were used to calculate the average EdU/DAPI ratio (×100%).

### *In vitro* cell migration and migration assays

Cells with applied genetic modifications were seeded into the upper surfaces of the “Transwell” chambers (BD Biosciences, San Jose, CA, USA ) at a density of 40, 000 cells per chamber (maintained in 300 μL serum-free medium [48]). FBS (12%) complete medium was filled into the lower chambers. Cells were allowed to migrate for 24h. Migrated cells on the lower surface of the chamber were stained and counted manually. For each condition, five repeated views were included to calculate the average number of migrated cells. For cell invasion assays, the chamber surface was always coated with “Matrigel” (Sigma).

### Caspase-3 activity assay

As described [27], total cellular protein lysates (30 μg for each treatment) were incubated with the 7-amino-4-trifluoromethylcoumarin (AFC)-conjugated caspase-3 substrate into the caspase assay buffer. After 3h of incubation, the AFC activity was examined by an Infinite 200 PRO reader at 400 nm excitation and 505 nm emission.

### Annexin V FACS

A431 cells with applied genetic modifications were initially seeded into six-well plates (1×10^5^ cells per dish) and cultured for 72h. Cells were then co-stained with Annexin V-FITC (15 μg/mL) and propidium iodide (PI, 15 μg/mL). Thereafter, a fluorescence-activated cell sorting (FACS, BD Biosciences) was applied to sort apoptotic cells (Annexin V^+/+^).

### TUNEL staining

Cells with applied genetic modifications were seeded into 96-well plates (4, 000 cells per well) and cultured for 72h. Cells were then stained with TUNEL and nuclear dye DAPI. TUNEL-positive nuclei ratio was calculated from five random views (1000 nuclei per treatment).

### DNA breaks

Skin SCC cells with applied genetic modifications were seeded into 96-well plates (4 × 10^3^ cells per well) and cultured for 48h. To test DNA breaks, a single strand DNA (ssDNA) ELISA kit (EMD Millipore, Burlington, MA, USA) was utilized. ELISA absorbance in each well was tested at 405 nm.

### Mitochondrial depolarization assay

Cells with applied genetic modifications were initially seeded into 12-well plates (4 ×10^4^ cells per well) for 48h and stained with JC-1 dye. The intensity of JC-1 green monomers was measured.

### Ceramide assay

Cells with applied genetic modifications were initially seeded into six-well plates (1×10^5^ cells per well) for 24h. The cellular ceramide content was examined through a protocol as previously described [49]. Ceramide content was expressed as fmol by nmol of phospholipid. Its level was normalized to the control.

### Ectopic overexpression of SphK1

The GV369 lentiviral construct encoding the UTR-null *SphK1* was received from Dr. Yao at Nanjing Medical University [22]. The construct was transduced to A439 cells with miR-6784 overexpression. Stable cells were selected by puromycin. SphK1 expression was verified by qPCR and Western blotting assays.

### CRISPR/Cas9-induced SphK1 knockout (KO)

A lentiCRISPR-Cas9-GFP SphK1 KO construct was from Dr. Yao at Nanjing Medical University [22]. A431 cells were cultured into six well-tissue plates at 50-60% confluence for 24h and then transfected with theSphK1-KO construct for another 48h. Afterwards, FACS-mediated GFP sorting was applied and the transfected cells were distributed into 96-well plates for another 72h. Single stable SphK1 KO A431 cells were further screened by qPCR and Western blotting assays. The empty vector, “Cas9-C” [22], was transfected to control A431 cells.

### Statistical analysis

Experiments were repeated for at least three times. Data were presented as mean ± standard deviation (SD). Statistics were analyzed by one-way ANOVA with SPSS software (21.0, Chicago, CA). To compare difference between two specific groups, a two tailed T Test was applied (Excel 2007). P<0.05 was considered for significant statistical difference.

## Supplementary Material

Supplementary Figure 1

## References

[1] Walls B, Jordan L, Diaz L, Miller R. Targeted therapy for cutaneous oncology: a review of novel treatment options for non-melanoma skin cancer: part II. J Drugs Dermatol. 2014; 13:955–58. 25116974

[2] Leiter U, Keim U, Garbe C. Epidemiology of Skin Cancer: Update 2019. Adv Exp Med Biol. 2020; 1268:123–39. 10.1007/978-3-030-46227-7_6 32918216

[3] Cleavenger J, Johnson SM. Non melanoma skin cancer review. J Ark Med Soc. 2014; 110:230–34. 24783362

[4] Karia PS, Han J, Schmults CD. Cutaneous squamous cell carcinoma: estimated incidence of disease, nodal metastasis, and deaths from disease in the United States, 2012. J Am Acad Dermatol. 2013; 68:957–66. 10.1016/j.jaad.2012.11.037 23375456

[5] Shida D, Takabe K, Kapitonov D, Milstien S, Spiegel S. Targeting SphK1 as a new strategy against cancer. Curr Drug Targets. 2008; 9:662–73. 10.2174/138945008785132402 18691013 PMC2674575

[6] Maceyka M, Harikumar KB, Milstien S, Spiegel S. Sphingosine-1-phosphate signaling and its role in disease. Trends Cell Biol. 2012; 22:50–60. 10.1016/j.tcb.2011.09.003 22001186 PMC3253987

[7] Pyne NJ, Pyne S. Sphingosine 1-phosphate and cancer. Nat Rev Cancer. 2010; 10:489–503. 10.1038/nrc2875 20555359

[8] Gangoiti P, Granado MH, Alonso A, Goñi FM, Gómez-Muñoz A. Implication of ceramide, ceramide 1-phosphate and sphingosine 1-phosphate in tumorigenesis. Transl Oncogenomics. 2008; 3:81–98. 21566746 PMC3022355

[9] Alemany R, van Koppen CJ, Danneberg K, Ter Braak M, Meyer Zu Heringdorf D. Regulation and functional roles of sphingosine kinases. Naunyn Schmiedebergs Arch Pharmacol. 2007; 374:413–28. 10.1007/s00210-007-0132-3 17242884

[10] Spiegel S, Milstien S. Sphingosine-1-phosphate: an enigmatic signalling lipid. Nat Rev Mol Cell Biol. 2003; 4:397–407. 10.1038/nrm1103 12728273

[11] Vadas M, Xia P, McCaughan G, Gamble J. The role of sphingosine kinase 1 in cancer: oncogene or non-oncogene addiction? Biochim Biophys Acta. 2008; 1781:442–47. 10.1016/j.bbalip.2008.06.007 18638570

[12] Kato K, Shimasaki M, Kato T, Segami N, Ueda Y. Expression of sphingosine kinase-1 is associated with invasiveness and poor prognosis of oral squamous cell carcinoma. Anticancer Res. 2018; 38:1361–68. 10.21873/anticanres.12359 29491060

[13] Tamashiro PM, Furuya H, Shimizu Y, Iino K, Kawamori T. The impact of sphingosine kinase-1 in head and neck cancer. Biomolecules. 2013; 3:481–513. 10.3390/biom3030481 24970177 PMC4030949

[14] Liu Z, Li P, Yang YQ, Cai S, Lin X, Chen MB, Guo H. I-BET726 suppresses human skin squamous cell carcinoma cell growth *in vitro* and *in vivo*. Cell Death Dis. 2020; 11:318. 10.1038/s41419-020-2515-z 32371868 PMC7200671

[15] Iqbal MA, Arora S, Prakasam G, Calin GA, Syed MA. MicroRNA in lung cancer: role, mechanisms, pathways and therapeutic relevance. Mol Aspects Med. 2019; 70:3–20. 10.1016/j.mam.2018.07.003 30102929

[16] Orang AV, Barzegari A. MicroRNAs in colorectal cancer: from diagnosis to targeted therapy. Asian Pac J Cancer Prev. 2014; 15:6989–99. 10.7314/apjcp.2014.15.17.6989 25227782

[17] Fu LL, Wen X, Bao JK, Liu B. MicroRNA-modulated autophagic signaling networks in cancer. Int J Biochem Cell Biol. 2012; 44:733–36. 10.1016/j.biocel.2012.02.004 22342941

[18] Calin GA, Croce CM. MicroRNA signatures in human cancers. Nat Rev Cancer. 2006; 6:857–66. 10.1038/nrc1997 17060945

[19] Konicke K, López-Luna A, Muñoz-Carrillo JL, Servín-González LS, Flores-de la Torre A, Olasz E, Lazarova Z. The microRNA landscape of cutaneous squamous cell carcinoma. Drug Discov Today. 2018; 23:864–70. 10.1016/j.drudis.2018.01.023 29317340

[20] Dumache R. Early diagnosis of oral squamous cell carcinoma by salivary microRNAs. Clin Lab. 2017; 63:1771–76. 10.7754/Clin.Lab.2017.170607 29226639

[21] Mei LL, Qiu YT, Zhang B, Shi ZZ. MicroRNAs in esophageal squamous cell carcinoma: potential biomarkers and therapeutic targets. Cancer Biomark. 2017; 19:1–9. 10.3233/CBM-160240 28269750 PMC13020702

[22] Yao C, Ruan JW, Zhu YR, Liu F, Wu HM, Zhang Y, Jiang Q. The therapeutic value of the SphK1-targeting microRNA-3677 in human osteosarcoma cells. Aging (Albany NY). 2020; 12:5399–410. 10.18632/aging.102961 32203055 PMC7138565

[23] Zhou Y, Han Y, Zhang Z, Shi Z, Zhou L, Liu X, Jia X. MicroRNA-124 upregulation inhibits proliferation and invasion of osteosarcoma cells by targeting sphingosine kinase 1. Hum Cell. 2017; 30:30–40. 10.1007/s13577-016-0148-4 27743351

[24] Zhang H, Wang Q, Zhao Q, Di W. MiR-124 inhibits the migration and invasion of ovarian cancer cells by targeting SphK1. J Ovarian Res. 2013; 6:84. 10.1186/1757-2215-6-84 24279510 PMC3879084

[25] Xia J, Wu Z, Yu C, He W, Zheng H, He Y, Jian W, Chen L, Zhang L, Li W. miR-124 inhibits cell proliferation in gastric cancer through down-regulation of SPHK1. J Pathol. 2012; 227:470–80. 10.1002/path.4030 22450659

[26] Agarwal V, Bell GW, Nam JW, Bartel DP. Predicting effective microRNA target sites in mammalian mRNAs. Elife. 2015; 4:e05005. 10.7554/eLife.05005 26267216 PMC4532895

[27] Liu Z, Wu G, Lin C, Guo H, Xu J, Zhao T. IGF2BP1 over-expression in skin squamous cell carcinoma cells is essential for cell growth. Biochem Biophys Res Commun. 2018; 501:731–38. 10.1016/j.bbrc.2018.05.057 29753746

[28] Perier C, Bové J, Vila M. Mitochondria and programmed cell death in Parkinson’s disease: apoptosis and beyond. Antioxid Redox Signal. 2012; 16:883–95. 10.1089/ars.2011.4074 21619488

[29] Chen M, Wang J. Initiator caspases in apoptosis signaling pathways. Apoptosis. 2002; 7:313–19. 10.1023/a:1016167228059 12101390

[30] Mullen TD, Obeid LM. Ceramide and apoptosis: exploring the enigmatic connections between sphingolipid metabolism and programmed cell death. Anticancer Agents Med Chem. 2012; 12:340–63. 10.2174/187152012800228661 21707511

[31] Ogretmen B, Hannun YA. Biologically active sphingolipids in cancer pathogenesis and treatment. Nat Rev Cancer. 2004; 4:604–16. 10.1038/nrc1411 15286740

[32] Ponnusamy S, Meyers-Needham M, Senkal CE, Saddoughi SA, Sentelle D, Selvam SP, Salas A, Ogretmen B. Sphingolipids and cancer: ceramide and sphingosine-1-phosphate in the regulation of cell death and drug resistance. Future Oncol. 2010; 6:1603–24. 10.2217/fon.10.116 21062159 PMC3071292

[33] Young MM, Kester M, Wang HG. Sphingolipids: regulators of crosstalk between apoptosis and autophagy. J Lipid Res. 2013; 54:5–19. 10.1194/jlr.R031278 23152582 PMC3520539

[34] Sheu JJ, Lee CC, Hua CH, Li CI, Lai MT, Lee SC, Cheng J, Chen CM, Chan C, Chao SC, Chen JY, Chang JY, Lee CH. LRIG1 modulates aggressiveness of head and neck cancers by regulating EGFR-MAPK-SPHK1 signaling and extracellular matrix remodeling. Oncogene. 2014; 33:1375–84. 10.1038/onc.2013.98 23624915

[35] Tamashiro PM, Furuya H, Shimizu Y, Kawamori T. Sphingosine kinase 1 mediates head & neck squamous cell carcinoma invasion through sphingosine 1-phosphate receptor 1. Cancer Cell Int. 2014; 14:76. 10.1186/s12935-014-0076-x 25197261 PMC4155094

[36] Schiefler C, Piontek G, Doescher J, Schuettler D, Mißlbeck M, Rudelius M, Haug A, Reiter R, Brockhoff G, Pickhard A. Inhibition of SphK1 reduces radiation-induced migration and enhances sensitivity to cetuximab treatment by affecting the EGFR / SphK1 crosstalk. Oncotarget. 2014; 5:9877–88. 10.18632/oncotarget.2436 25245676 PMC4259444

[37] Zhao X, He W, Li J, Huang S, Wan X, Luo H, Wu D. MiRNA-125b inhibits proliferation and migration by targeting SphK1 in bladder cancer. Am J Transl Res. 2015; 7:2346–54. 26807182 PMC4697714

[38] Li J, Wu H, Li W, Yin L, Guo S, Xu X, Ouyang Y, Zhao Z, Liu S, Tian Y, Tian Z, Ju J, Ni B, Wang H. Downregulated miR-506 expression facilitates pancreatic cancer progression and chemoresistance via SPHK1/Akt/NF-κB signaling. Oncogene. 2016; 35:5501–14. 10.1038/onc.2016.90 27065335 PMC5078861

[39] Xiang T, Bai JY, She C, Yu DJ, Zhou XZ, Zhao TL. Bromodomain protein BRD4 promotes cell proliferation in skin squamous cell carcinoma. Cell Signal. 2018; 42:106–13. 10.1016/j.cellsig.2017.10.010 29050985

[40] Xu HB, Zheng YF, Wu D, Li Y, Zhou LN, Chen YG. microRNA-1203 targets and silences cyclophilin D to protect human endometrial cells from oxygen and glucose deprivation-re-oxygenation. Aging (Albany NY). 2020; 12:3010–24. 10.18632/aging.102795 32041924 PMC7041737

[41] Tang XF, Liu HY, Wu L, Li MH, Li SP, Xu HB. Ginseng Rh2 protects endometrial cells from oxygen glucose deprivation/re-oxygenation. Oncotarget. 2017; 8:105703–13. 10.18632/oncotarget.22390 29285285 PMC5739672

[42] Yang L, Zheng LY, Tian Y, Zhang ZQ, Dong WL, Wang XF, Zhang XY, Cao C. C6 ceramide dramatically enhances docetaxel-induced growth inhibition and apoptosis in cultured breast cancer cells: a mechanism study. Exp Cell Res. 2015; 332:47–59. 10.1016/j.yexcr.2014.12.017 25576381

[43] Li KR, Zhang ZQ, Yao J, Zhao YX, Duan J, Cao C, Jiang Q. Ginsenoside Rg-1 protects retinal pigment epithelium (RPE) cells from cobalt chloride (CoCl2) and hypoxia assaults. PLoS One. 2013; 8:e84171. 10.1371/journal.pone.0084171 24386346 PMC3873980

[44] Chen ZJ, Rong L, Huang D, Jiang Q. Targeting cullin 3 by miR-601 activates Nrf2 signaling to protect retinal pigment epithelium cells from hydrogen peroxide. Biochem Biophys Res Commun. 2019; 515:679–87. 10.1016/j.bbrc.2019.05.171 31178131

[45] Liu YY, Chen MB, Cheng L, Zhang ZQ, Yu ZQ, Jiang Q, Chen G, Cao C. microRNA-200a downregulation in human glioma leads to Gαi1 over-expression, Akt activation, and cell proliferation. Oncogene. 2018; 37:2890–902. 10.1038/s41388-018-0184-5 29520106

[46] Wang R, Zhang S, Chen X, Li N, Li J, Jia R, Pan Y, Liang H. CircNT5E acts as a sponge of miR-422a to promote glioblastoma tumorigenesis. Cancer Res. 2018; 78:4812–25. 10.1158/0008-5472.CAN-18-0532 29967262

[47] Wang K, Long B, Liu F, Wang JX, Liu CY, Zhao B, Zhou LY, Sun T, Wang M, Yu T, Gong Y, Liu J, Dong YH, et al. A circular RNA protects the heart from pathological hypertrophy and heart failure by targeting miR-223. Eur Heart J. 2016; 37:2602–11. 10.1093/eurheartj/ehv713 26802132

[48] Sun J, Huang W, Yang SF, Zhang XP, Yu Q, Zhang ZQ, Yao J, Li KR, Jiang Q, Cao C. Gαi1 and Gαi3mediate VEGF-induced VEGFR2 endocytosis, signaling and angiogenesis. Theranostics. 2018; 8:4695–709. 10.7150/thno.26203 30279732 PMC6160771

[49] Gong L, Yang B, Xu M, Cheng B, Tang X, Zheng P, Jing Y, Wu GJ. Bortezomib-induced apoptosis in cultured pancreatic cancer cells is associated with ceramide production. Cancer Chemother Pharmacol. 2014; 73:69–77. 10.1007/s00280-013-2318-3 24190701

