# Supplementary material for: SphK1-targeted miR-6784 inhibits functions of skin squamous cell carcinoma cells
Source: Aging (Albany NY). 2021 Jan 19;13(3):3726–41. doi: 10.18632/aging.202336 (PMC7906188; doi:10.18632/aging.202336)
Supplement: Supplementary Figure 1 [file aging-13-202336-s001.pdf]

SUPPLEMENTARY FIGURE

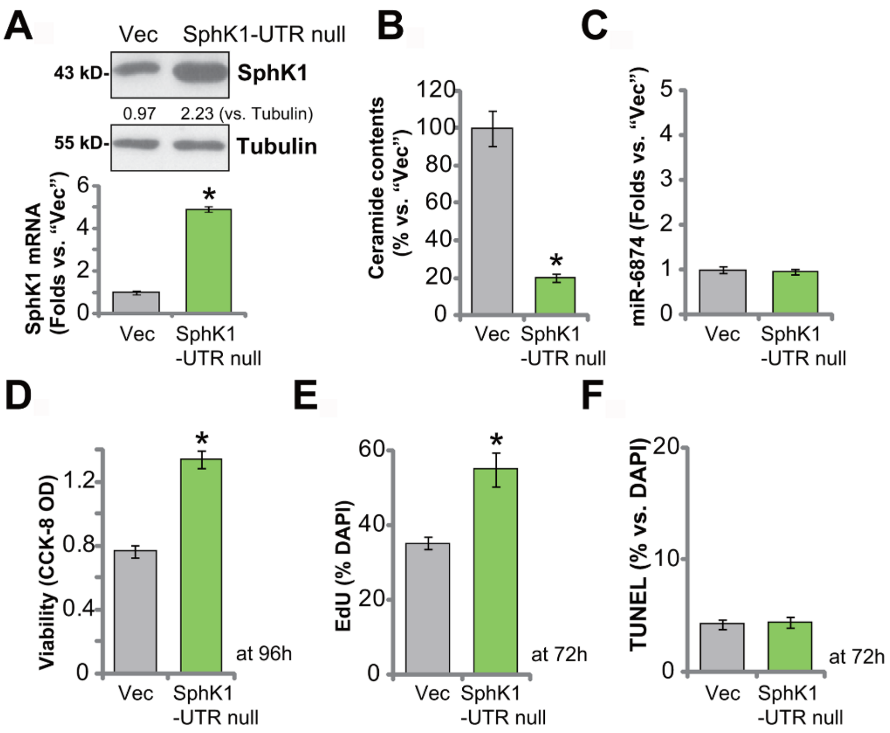

Supplementary Figure 1. A431 cells were transduced with an UTR-null SphK1 construct ("SphK1-UTR null") or the empty vector ("Vec"). Stable cells were established with puromycin selection. Expression SphK1/2 and miR-6784 was shown (**A**, **C**). Cellular ceramide contents were tested (**B**); Cells were further cultured for applied time periods, cell viability, proliferation, and apoptosis were tested by CCK-8 (**D**), EdU staining (**E**), and TUNEL staining (**F**) assays, respectively. Data were presented as mean ± standard deviation (SD, n=5). Experiments in this study were repeated three times with similar results obtained. *p< 0.05 *vs.* "Vec" cells.
